# Urine interleukin-18 in prediction of acute kidney injury: a systemic review and meta-analysis

**DOI:** 10.1007/s40620-014-0113-9

**Published:** 2014-06-05

**Authors:** Xin Lin, Jing Yuan, Yingting Zhao, Yan Zha

**Affiliations:** 1 Department of Nephrology, People’s Hospital of Guizhou Province, No. 83, Zhongshan East Road, Guiyang, 550002 Guizhou People’s Republic of China; 2 Department of Nephrology, Nanfang hospital, Southern Medical University, Guangzhou, People’s Republic of China

**Keywords:** Interleukin-18, Acute kidney injury, Predictive value, Systemic review

## Abstract

**Background:**

Interleukin-18 (IL-18) mediates ischemic acute tubular necrosis; it has been proved as a rapid, reliable, and affordable test marker for the early detection of acute kidney injury (AKI), but its predictive accuracy varies greatly.

**Methods:**

MEDLINE and EMBASE, Cochrane Library, Ovid, and Springerlink (from inception to November 15, 2013) were searched for relevant studies (in English) investigating diagnostic accuracy of urine IL-18 to predict AKI in various clinical settings. The text index was increasing or increased urine IL-18 level and the main outcome was the development of AKI, which was primarily based on serum creatinine level [using risk, injury, failure, loss and end-stage renal disease (RIFLE), acute kidney injury network, or modified pediatric RIFLE criteria in pediatric patients]. Pooled estimates of diagnostic odds ratio (OR), sensitivity and specificity were calculated. Summary receiver operating characteristic curves were used to calculate the measures of accuracy and Q point value (Q*). Remarkable heterogeneity was explored further by subgroup analysis based on the different clinical settings.

**Results:**

We analyzed data from 11 studies of 3 countries covering 2,796 patients. These studies were marked by limitations of threshold and non-threshold effect heterogeneity. Across all settings, the diagnostic OR for urine IL-18 level to predict AKI was 5.11 [95 % confidence interval (CI) 3.22–8.12], with sensitivity and specificity respectively at 0.51 and 0.79. The area under the ROC curve of urine IL-18 level to predict AKI was 0.77 (95 % CI 0.71–0.83). Subgroup analysis showed that urine IL-18 level in pediatric patients (<18 years) and early AKI predictive time (<12 h) were more effective in predicting AKI, with diagnostic ORs of 7.51 (2.99–18.88), 8.18 (2.19–30.51), respectively.

**Conclusion:**

Urine IL-18 holds promise as a biomarker in the prediction of AKI but has only moderate diagnostic value.

## Introduction

As an abrupt or rapid decline in renal filtration function, acute kidney injury (AKI) is a common condition associated with significant morbidity and mortality. In critically ill patients, those with AKI usually present a worse clinical outcome than their non-AKI counterparts [1]. Although regarded as the standard indicators of kidney function loss, serum creatinine (sCr) level and urine output are recognized as having limitations. On the one hand, sCr cannot accurately reflect the glomerular filtration rate (GFR) in a patient with unsteady state, and urine output is easily affected by water intake and the primary water load of the body [2]. On the other hand, sCr and urine output have limited sensitivity and specificity, and a delayed response to kidney impairment. All these factors suggest that sCr and urine output are not appropriate markers in the early detection of AKI. Thus, an accurate and timely biomarker to predict AKI onset or progression after renal insult is urgently needed.

Interleukin-18 (IL-18), a member of the IL-1 family of cytokines, is synthesized as an inactive 23-kDa precursor by several tissues including monocytes, macrophages, and proximal tubular epithelial cells, and is processed into an active 18.3 kDa cytokine by caspase-1 [3]. It has been demonstrated in some animal studies [4] as a mediator of renal ischemia–reperfusion injury, inducing acute tubular necrosis, and neutrophil and monocyte infiltration of the renal parenchyma. More recently, numerous clinical studies have focused on the diagnostic accuracy of IL-18 level in predicting AKI [5–7]. With the evidence accumulating, contradictory results have raised concerns about the predictive value of AKI across various settings. Thus, we performed a systematic review and meta-analysis to investigate the diagnostic accuracy of IL-18 level for predicting AKI. Since there is no clear consensus about the appropriate cutoff level of IL-18 to predict AKI and different thresholds have been reported by different studies, the summary receiver operating characteristic (sROC) curve was delineated for the meta-analysis.

## Methods

### Data sources and search strategy

A computer-based search was performed in MEDLINE, EMBASE, Cochrane Library, Ovid, and Springerlink databases, from inception until November 15, 2013, to identify potentially relevant articles. The search strategy consisted of terms related to AKI (“acute kidney injury” and “acute renal failure”) combined with the term “interleukin-18/interleukin 18/IL-18”. To ensure all articles were located, a secondary search of the articles already retrieved was undertaken, reviewing their reference lists. The preferred reporting items for systematic reviews and meta-analysis (PRISMA) guidelines for the conduct of meta-analyses were followed [8].

### Study selection

The titles and abstracts were examined independently by two of the authors (X. L. and J. Y.) to ascertain their relevance and inclusion for further analysis. Any disagreements were settled by consensus using a third opinion (Y. Z.). Inclusion criteria were that studies investigated the diagnostic accuracy of urinary IL-18 to predict AKI and had data which could be extracted into a 2 × 2 table or complete data which could be obtained from the corresponding author by e-mail. The search was restricted to human subjects. Only papers in English were eligible.

### Data extraction

The following variables were recorded or recalculated: study population, sample size, age, sex, baseline IL-18 level, cutoff value for urine IL-18, AKI definition, number of patients who developed AKI, timing of obtaining the specimen, specificity, sensitivity, and area under the ROC curve (AUROC) with 95 % confidence interval (CI). Data for IL-18 sample storage and detection method were also collected. Two reviewers independently excluded articles on the basis of the title and abstract following a custom-made standardized table. The true-positive, true-negative, false-positive, and false-negative results of each included study were quantified. Quality assessment was performed using the Quality Assessment of Diagnostic Accuracy Studies (QUADAS) tool [9] and independently by the authors (X. L. and J. Y.).

### Statistical analysis

The meta-analysis was conducted using sROC curve as described by Rosman [10] and the computation was performed using Meta-Disc1.4. If multiple cutoff points were adopted in any study, the cutoff point (test threshold), sensitivity and specificity in this analysis were selected according to the Youden index. Heterogeneity was quantitatively assessed using the Q statistic. Fixed or random effects models (FEM or REM) were used on the basis of heterogeneity. We performed subgroup analysis to explore the remarkable heterogeneity in the light of different clinical settings.

## Results

### Search results and study characteristics

**Fig. 1:**
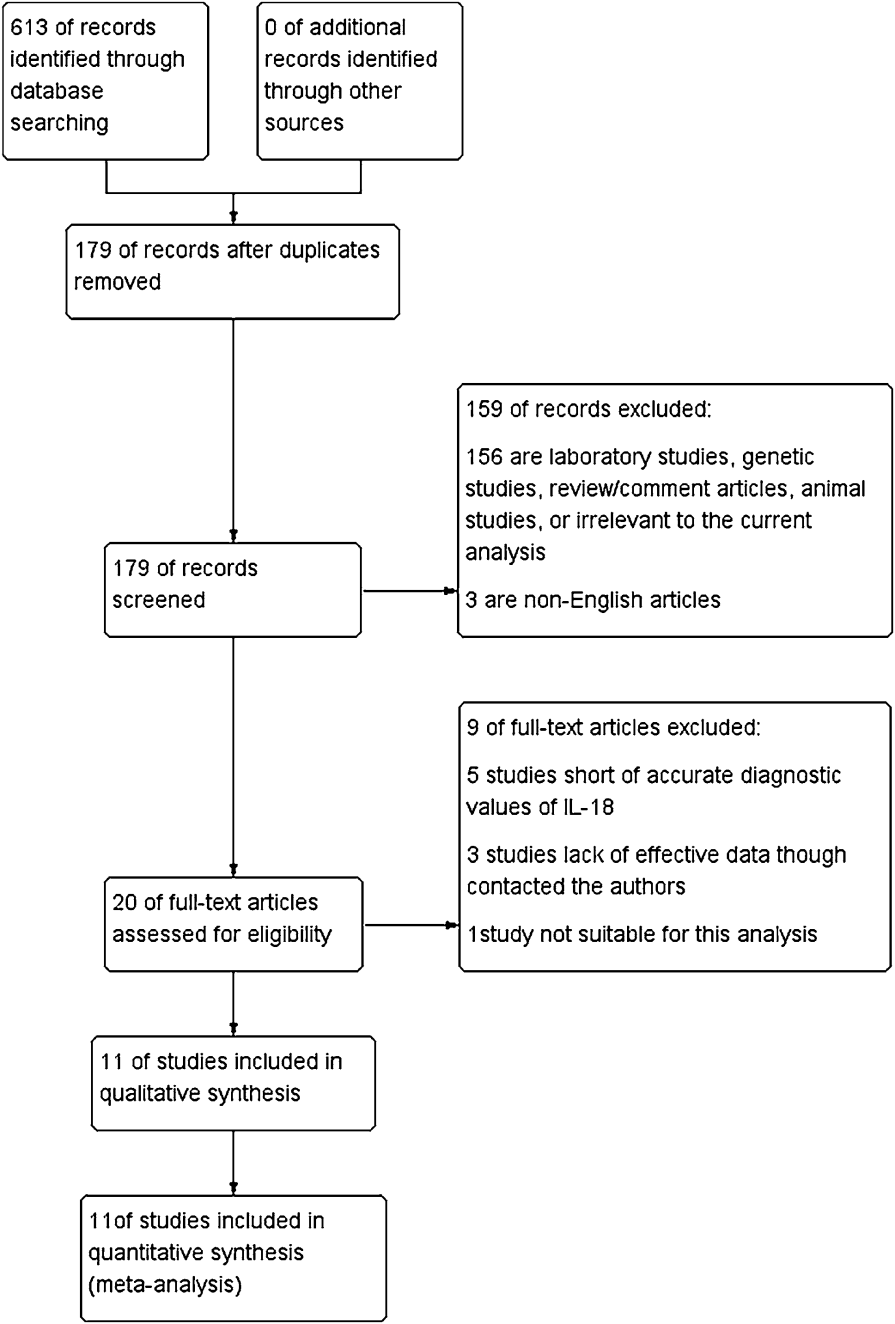
Flowchart representing study selection for systematic review of urine IL-18 as a diagnostic marker for AKI. *IL* interleukin, *AKI* acute kidney injury

Of 179 primary articles searched, 156 were excluded on the basis of abstract and/or title because they were laboratory studies, genetic studies, review/comment articles, animal studies, or irrelevant to the current analysis; 3 were excluded because non-English articles. Of the 20 remaining articles, 5 studies with accurate diagnostic values of IL-18 missing and 3 studies lacking effective data (though we had contacted the corresponding authors to request the missing information) were excluded. As a result, 11 studies [6, 7, 11–19] met the inclusion criteria and were selected for the analysis (Fig. 1).

Characteristics of the included trials are shown in Table 1. Two come from China [6, 11], seven from the USA [7, 13, 14, 16–19], one from Taiwan, China [12], and one from Australia [15]. Overall, 2,796 patients were comprised, ranging from newborn to elderly age. Sample sizes were >100 in 7 studies and >1,000 in 1 [14]. Most studies were conducted on critical patients, including those with cardiac surgery (cardiopulmonary bypass) and transplantation, as well as various intensive care patients. Five studies [6, 11, 13, 17, 18] focused on pediatric patients. Males in all studies represented >50 % of the study population. Eight studies defined AKI using acute kidney injury network (AKIN) and/or risk, injury, failure, loss and end-stage renal disease (RIFLE) criteria, two [11, 17] using modified pediatric RIFLE, and one [16] using sCr level which, however, rose to ≥50 % of baseline in the first 72 h. Ten studies harnessed urine IL-18 concentration as the cutoff value and two [11, 15] the ratio of urine IL-18 to urine-creatinine concentration.

**Table 1:** Characteristics of included studies

Study (first author + year)	Country of origin	Study population	Mean age (years)	Men (%)	Sample size	Number (%) of AKI	Mean baseline IL-18 (pg/ml)	Definition of AKI	IL-18 Assay	Sample storage (°C)
Zheng et al. [6]	China	Children with CHD or undergoing CPB surgery	Non-AKI 11.4 (2.2–47.0) AKI 5.9 (0.6–44.5)^a^	39 (67.2)	58	29 (50)	Non-AKI 7.9 (3.8–23.1) AKI 17.6 (7.2–41.5)	SCr↑ ≥0.3 mg/dl or ≥50 % baseline, urine ≤0.5 ml/kg/6 h AKIN criteria	ELISA	–80
Sirota et al. [7]	USA	Orthotopic liver transplantation patients	56.1 ± 6.8	27 (67.5)	40	7 (17.5)	AKI 0 (0–18.57) Non-AKI 0 (0–200.10)	SCr↑ ≥50 % baseline, RIFLE and AKIN criteria	ELISA	–80
Li et al. [11]	China	Non-septic critically ill neonates	34.1 ± 3.2^b^	34 (54.8)	62	11 (17.7)	/	SCr >1.5 mg/dl on the first 3 days of life, after the first 3 days of life, a ≥25 % decrease in eCCl. Modified pediatric RIFLE	ELISA	–80
Chen et al. [12]	Taiwan, China	CCU patients	66 ± 1	113 (75)	150	43 (28.7)	71 ± 5	AKIN criteria	ELISA	–80
Parikh et al. [13]	USA	Pediatric patients with congenital cardiac lesions surgery	3.8 ± 4.5	171 (55)	311	53 (17.0)	/	Receipt of acute dialysis during the entire hospital stay or sCr double baseline. RIFLE R or AKIN stage 2	ELISA	–80
Parikh et al. [14]	USA	Cardiac surgery adults at high risk for AKI	71 ± 10	826 (68)	1219	60 (4.9)	/	Receipt of acute dialysis during the entire hospital stay or sCr double baseline. RIFLE R or AKIN stage 2	ELISA	–80
Endre et al. [15]	Australia	ICU admission	60 ± 17	367 (70.2)	523	147 (28.1)	73 ± 340^c^	SCr↑ ≥0.3 mg/dl or 50 % baseline AKIN48 or RIFLE 24 criteria	ELISA	–80
Liangos et al. [16]	USA	Patients undergoing on-pump cardiac surgery	68 ± 11	74 (72)	103	13 (12.6)	/	Scr↑ ≥50 % baseline in the first 72 h following termination of CPB	ELISA	–80
Washburn et al. [17]	USA	PICU children who received mechanical ventilation	6.5 ± 6.4	73 (53)	137	103 (75.2)	179.0 ± 337.9	Paediatric modified RIFLE	ELISA	–80
Parikh et al. [18]	USA	Cardiac surgery children	3.4 ± 5.3	30 (54.5)	55	20 (36.4)	1.65 ± 1.01	SCr↑ ≥50 % baseline	ELISA	–80
Parikh et al. [19]	USA	ARDS and ALI patient	50.2 ± 17.0	72 (52.2)	138	52 (37.7)	AKI 104 (0–955) Control 0 (0–173)	SCr↑ ≥50 % baseline	ELISA	–80

*AKI* acute kidney injury, *AKIN* acute kidney injury network, *ELISA* enzyme-linked immunosorbent assay, *CHD* congenital heart disease, *CPB* cardiopulmonary bypass, *sCr* serum creatinine, *RIFLE* risk, injury, failure, loss and end-stage renal disease, *eCCl* estimated Cr clearance, *CCU* coronary care unit, *ICU* intensive care unit, *PICU* pediatric intensive care unit, *ARDS* acute respiratory distress syndrome, *ALI* acute lung injury

^a^Months; ^b ^Gestational age, weeks; ^c ^(pg/ml)/mmol/l Cr

### Quality assessment

Table 2 lists the methodological quality assessment of the included 11 studies. Ten studies enrolled single or multicenter consecutive patients prospectively, one [7] was retrospective and prospective in design. The study by Chen et al. [12] did not define inclusion and exclusion criteria clearly. The reference standards used in the afore-mentioned studies were creatinine based, which currently is the most widely used standard for evaluating kidney function. The conditions of follow-up were reported in the 10 prospective cohort studies; there were no selective losses in them. Overall, the quality of the studies was suboptimal, without sufficient information to assess all the bias, and uninterpretable, indeterminate or intermediate index test results were not reported. The quality assessment was performed independently by the authors (X. L. and J. Y.).

**Table 2:** Quality assessment of individual studies

Study	Study design	Spectrum bias^a^	Eligibility criteria clearly defined	Appropriate reference standard	Differential verification bias^b^	Index test and reference standard sufficiently described	Selective loss during followup^c^	Important confounders identified
Zheng et al. [6]	Prospective study	No	Yes	Yes	Yes	Yes	No	Yes
Sirota et al. [7]	Retrospective and prospective study	No	Yes	Yes	Yes	Yes	–	Yes
Li et al. [11]	Prospective study	No	Yes	Yes	Yes	Yes	No	Yes
Chen et al. [12]	Prospective study	No	No	Yes	Yes	Yes	No	Yes
Parikh et al. [13]	Prospective, multicenter cohort study	No	Yes	Yes	Yes	Yes	No	Yes
Parikh et al. [14]	Prospective, multicenter cohort study	No	Yes	Yes	Yes	Yes	No	Yes
Endre et al. [15]	Prospective observational study	No	Yes	Yes	Yes	Yes	No	Yes
Liangos et al. [16]	Prospective cohort study	No	Yes	Yes	Yes	Yes	No	Yes
Washburn et al. [17]	Prospective study	No	Yes	Yes	Yes	Yes	No	Yes
Parikh et al. [18]	Prospective study	No	Yes	Yes	Yes	Yes	No	Yes
Parikh et al. [19]	Prospective study	No	Yes	Yes	Yes	Yes	No	Yes

^a^This item is labeled “No” if the spectrum of patients was representative of patients who received the test in practice, otherwise it is labeled “Yes”

^b^This item is labeled “No” when all patients received the same reference standard, otherwise it is labeled “Yes”

^c^This item is labeled “Yes” if patients who were lost to follow-up differed systematically from those who remained, otherwise it is labeled “No”

“—” This item is not applicable if the study was retrospective in design

### Data extraction and calculation

As the respective number of true-positive, false-positive, true-negative, and false-negative results was not provided by the studies, these indexes were calculated from available sensitivity, specificity, and sample size values. Results are reported in Table 3 which enumerates specimen obtaining times and AKI predictive times of each study. Cutoff and AUROC values are also included in the table. As can be seen, cutoff values for urine IL-18 varied across studies.

**Table 3:** Sensitivity and specificity of individual studies for urine IL-18 to predict AKI

Study	Time of obtaining specimen	TP	FP	FN	TN	Cutoff value (pg/ml)	Sen*s*itivity (%)	Specificity (%)	AUROC (95 % CI)	Assess time (h)
Zheng et al. [6]	0, 4, 6, 12, and 24 h after the initiation of CPB	28	11	1	18	49	96.60	62.10	0.835 (0.729–0.940)	4
Sirota1 et al. [7]	24 h after orthotopic liver transplantation	5	7	2	26	/	72	79	0.749	24
Li et al. [11]	48 h admitted	7	4	4	47	1,800^a^	64	92	0.72 (0.52–0.93)	48
Chen et al. [12]	Admission	22	17	22	90	70	50	84	0.621 (0.504–0.738)	48
Parikh et al. [13]	Admission	37	83	16	175	125	69	68	0.72 (0.64–0.80)	48
Parikh et al. [14]	Admission, every 6 h	32	209	28	950	60	54	82	0.74 (0.66–0.81)	48
Endre et al. [15]	Admission	50	83	97	293	36^b^	34	78	0.62 (0.56–0.67)	0
Liangos et al. [16]	2 h post-cardiopulmonary bypass	10	31	3	59	92	75	66	0.66 (0.49–0.83)	2
Washburn et al. [17]	2 PM each day	39	7	64	27	75	38	78	0.54 (0.31–0.77)	24
		55	10	48	24	75	53	71	0.61 (0.43 –0.78)	48
Parikh et al. [18]	Every 2 h for the first 12 h and then once every 12 h	5	1	15	34	50	25	97	0.61	4
		10	2	10	33	50	50	94	0.75	12
		12	4	8	31	20	60	89	0.73	24
Parikh et al. [19]	Days 0, 1, and 3	38	29	14	57	25	74	66	0.731	24

*IL* interleukin, *AKI* acute kidney injury, *AUROC* area under the receiver operating characteristic curve, *CI* confidence interval, *CPB* cardiopulmonary bypass, *FN* false-negative, *FP* false-positive, *TN* true-negative, *TP* true-positive

^a^pg/mg uCr

^b^(pg/ml)/mmol/l Cr

### Diagnostic value of urine IL-18 in AKI prediction

**Fig. 2:**
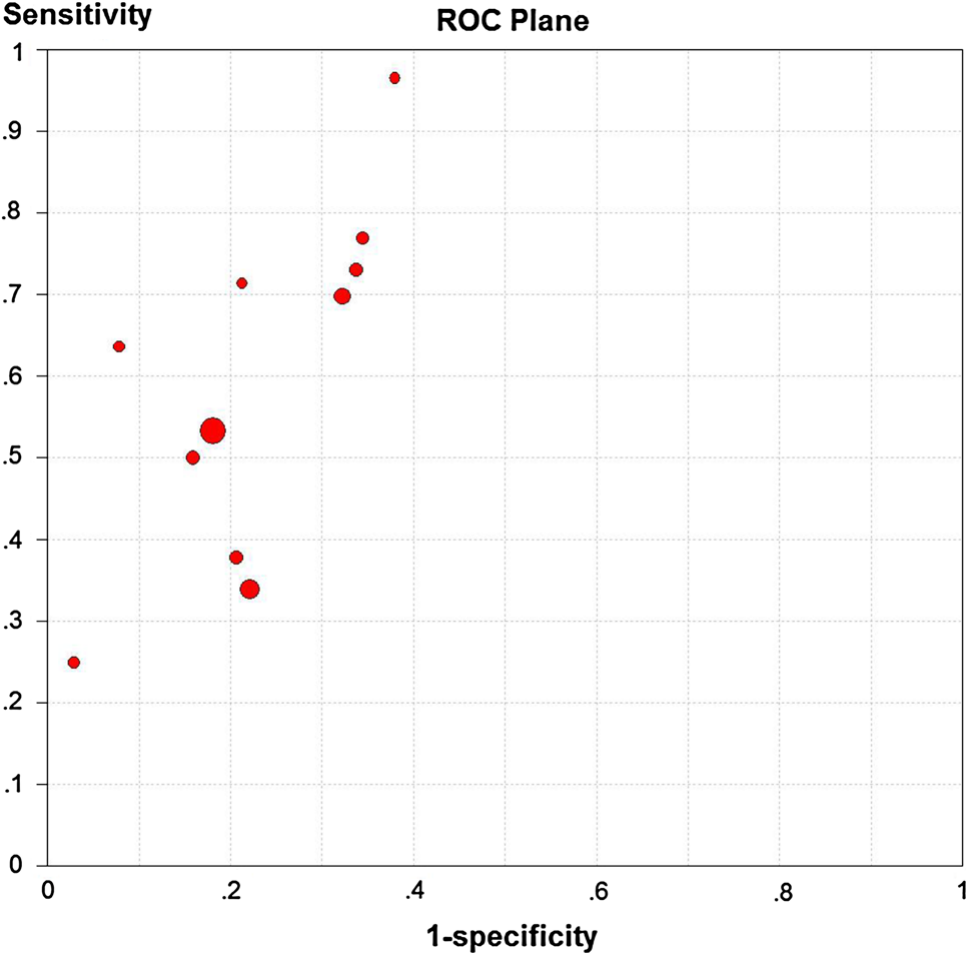
The distribution of accurate estimator in sROC curve floor plan. *sROC* summary receiver operating characteristic

The distribution of accurate estimator in sROC curve floor plan and the Spearman correlation coefficient (r = 0.764, p = 0.006) of logarithmic sensitivity and logarithm (1-specificity) showed that there was a threshold effect in different studies (Fig. 2; Table 4).

**Table 4:** Analysis of diagnostic threshold

Spearman correlation coefficient: 0.764, *p* value = 0.006				
Logit (TPR) vs. logit (FPR)				
Moses’ model (D = a + bS)				
Weighted regression (inverse variance)				
Var	Coeff.	Std. error	T	*p* value
a	1.791	0.254	7.061	0.0001
b(1)	0.224	0.157	1.428	0.1870

Tau-squared estimate = 0.1904 (convergence is achieved after 6 iterations)

Restricted maximum likelihood estimation (REML)

No. studies = 11 add 1/2 to all cells of the studies with zero

**Fig. 3:**
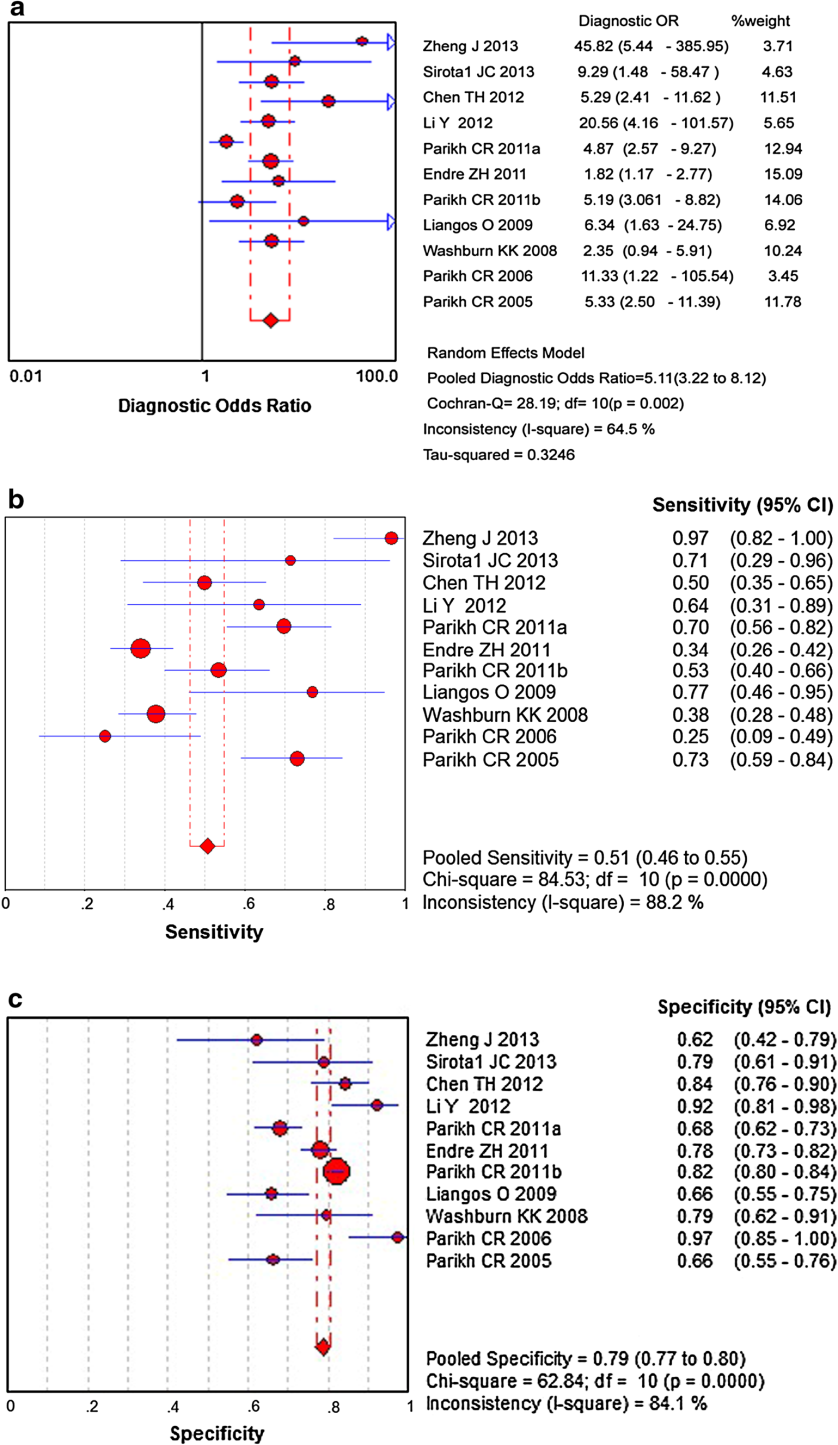
Forest plots and pooled estimates of **a** diagnostic odds ratio (OR), **b** sensitivity, and **c** specificity

**Fig. 4:**
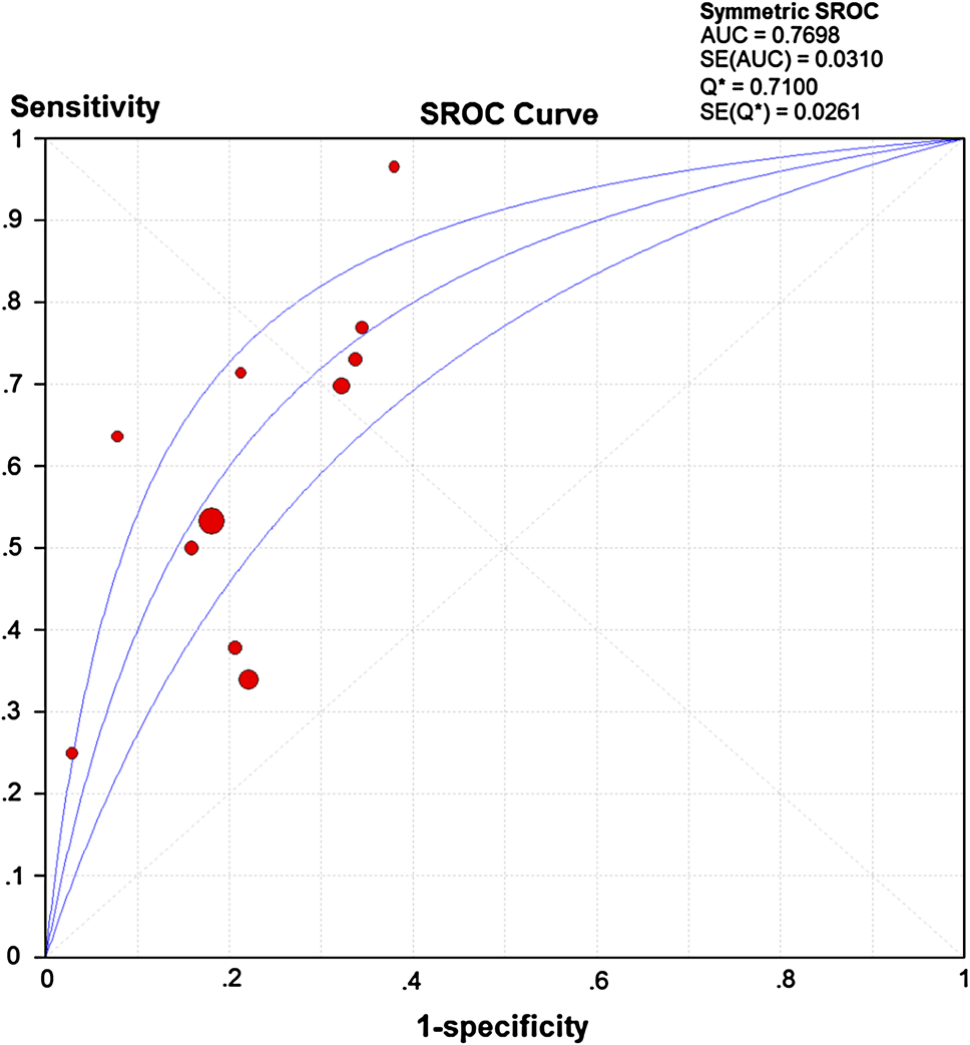
The fitting curve of sROC

Figure 3 shows the forest plots and pooled estimates of sensitivity, specificity and diagnostic odds ratios (ORs) respectively. We found a diagnostic OR of 5.11 (95 % CI 3.22–8.12) for urine IL-18 level to predict AKI (Cochran-Q = 28.19, p = 0.0017) with a sensitivity and specificity respectively of 0.51 (heterogeneity Chi-squared 84.53, p < 0.001) and 0.79 (heterogeneity Chi-squared 62.84, p < 0.001) (Fig. 3). Significant non-threshold effect heterogeneity was also disclosed across these studies. As there was no statistically significant between b and 0 (p = 0.1870), the Mantel–Haenszel model was utilized to draw the fitting curve of sROC (Fig. 4). The pooled AUROC was 0.77 (95 % CI 0.71–0.83, Q = 0.71).

Subgroup analysis (Table 5) showed that urine IL-18 level in pediatric patients (<18 years) was more effective in predicting AKI, with a diagnostic OR of 7.510 (95 % CI 2.988–18.875) compared to 4.652 (2.710–7.987) for the adult group (p = 0.334); the early AKI predictive time (<12 h) subgroup displayed the highest diagnostic OR of 8.176 (95 % CI 2.191–30.507) among the three subgroups (<12, 24 and 48 h, p = 0.037). The pooled estimate diagnostic OR of the admission subgroup was 3.81 (95 % CI 2.08–6.99) and that of the subgroup for other times 7.16 (3.62–14.18). There were no significant differences between the two (p = 0.388). There were significant differences (p = 0.008) between cardiac surgery patients and patients in intensive care unit or coronary care unit patients in the subgroup analysis, with diagnostic ORs of 5.28 (3.59–7.76) and 5.31 (2.59–10.87), respectively.

**Table 5:** Pooled diagnostic accuracy of IL-18 in various settings

Subgroup factors	Subgroup criteria	Reference numbers	Q value	P value	I^2^ (%)	Hierarchical summary OR (95 % CI)	P value for between subgroups
Age	<18 years	5	10.23	0.037	60.9	7.51 (2.99–18.88)	0.334
	≥18 years	6	15.54	0.008	67.8	4.32 (2.48–7.51)	
Predictive time	≤12 h	5	18.27	0.001	78.1	8.18 (2.19–30.51)	0.037
	24 h	4	4.48	0.214	33.0	5.07 (2.58–9.96)	
	48 h	5	5.04	0.283	20.7	4.95 (3.39–7.24)	
Obtaining specimen	Admission	4	13.4	0.004	77.6	3.81 (2.08–6.99)	0.388
	Other times	7	10.38	0.109	42.4	7.16 (3.62–14.18)	
Patients	Cardiac surgery	4	0.59	0.90	0.01	5.28 (3.593–7.762)	0.008
	Other patients	7	23.28	0.001	74.2	5.31 (2.59–10.87)	

*OR* odds ratio, *CI* confidence interval

## Discussion

The current study presents a meta-analysis of urine IL-18 for predicting AKI development via the sROC analysis approach, both overall and across a range of subgroups. Studies committed to direct comparisons between urine IL-18 and the “gold standard” sCr were included. The finding derived is consistent with previous studies, which lends more force to the use of urine IL-18 as a marker of AKI in clinical practice. The results of this meta-analysis indicate that urine IL-18 had a pooled diagnostic OR of 5.11 and the estimated area under the curve (AUC) of the mean ROC plot was 0.77 (Q = 0.71), with a high heterogeneity in pooled sensitivity and specificity. This suggests that urine IL-18 has a higher chance of early detecting an AKI compared to sCr, but the application of this biomarker in the diagnosis of AKI should be limited to a certain range in the light of limitations intrinsic to such studies. Since pooled results may increase statistical power and lead to more precise estimates of a treatment effect and the pooled random or fixed effect models reflect the between-study variance [20], this may shed light on larger populations.

Due to the presence of a threshold effect, we used sROC curve fitting, the area under ROC and Q index, to merge the data. In the studies included, cutoff values were expressed in two types, urinary concentration or ratio of urine IL-18 and creatinine concentration, and identified as varying across studies (11.25–125 pg/ml). Such differences can be explained by: (1) use of different reagents in these studies though the assay methods were enzyme-linked immunosorbent assay (ELISA); and (2) differences in clinical settings and study population. Therefore, it might be necessary for each center using urine IL-18 level for early AKI diagnosis to define a specific reference range and cutoff value for each clinical setting.

In addition, a significant non-threshold effect heterogeneity exists across these studies. Since these studies were based on different institutions across the world, heterogeneity was inevitable concerning differences in AKI definitions, AKI settings, times for obtaining specimen, and experimental groups admitted to assess the predictive value of urine IL-18. We attempted to use meta-regression to explore the sources of heterogeneity, but failed. So subgroup analysis was performed and proved that urine IL-18 level in pediatric patients and the early AKI predictive time group (<12 h) was more effective in predicting AKI, which might principally account for the heterogeneity. Nevertheless, as the kidney undergoes growth and maturation in neonates who manifest different physiological states due to the abrupt changes at birth, many risk factors are correlated with AKI and the timing of kidney injury remains unknown [21]. The metabolic capacity and compensatory ability of neonates and children are also different from those of adults [22]. Subgroup analysis of age should be more specific if more clinical trials are conducted. Regarding AKI definition, AKIN, RIFLE, AKIN and/or RIFLE, and modified pediatric RIFLE criteria were adopted in the different included studies. The pooled diagnostic OR of AKI within 12 h was greater than that of 24 or 48 h in subgroup analysis. This is useful in clinical processing since preventive strategies can be formulated if AKI can be predicted in advance.

Using sCr level as the “gold standard” of diagnosis of AKI is another limitation of such studies because sCr is not an ideal marker of early loss of glomerular filtration or kidney injury [23]. The best method is to use radio-labeled tracer clearances to define AKI. However, its use in routine clinical practice is restrained because it is invasive, time-consuming and radioactive.

Another limitation lies in IL-18 itself. Cross-sectional studies indicate that urinary IL-18 levels are markedly elevated in patients with acute tubular necrosis compared with healthy controls and a variety of other renal pathologies, including urinary tract infection, chronic renal insufficiency, and pre-renal azotemia [24]. Therefore, the pathophysiology of IL-18 still remains incompletely understood and the true role of IL-18 may be as a mediator of specific injury subtypes rather than as a marker of injury. Though IL-18 can be induced in the proximal tubule after AKI, and released into urine after cleavage by caspase-1 [25, 26], it can also be derived from lung injury and myocardial ischemia, etc. Thus, further studies are required to understand these differences.

Generally speaking, the cost of a single creatinine test is less than a dollar, while that of IL-18 is five times higher. In the course of AKI, biochemical indices need to be monitored and detected many times; given the modest clinical value of IL-18 and its high cost, serum creatinine concentration and change might still be a good indicator rather than IL-18.

## Conclusion

In conclusion, this meta-analysis included more than 2,700 patients from 11 studies. The result shows that urine IL-18 level is of diagnostic value for AKI. The diagnostic accuracy for AKI tends to be more effective in pediatric patients and early AKI predictive time. To improve the diagnostic value of urine IL-18, more appropriately designed investigations (e.g. with randomized design and eliminating potential confounders) should be performed.

## References

[1] Fuchs L, Lee J, Novack V (2013). Severity of acute kidney injury and two-year outcomes in critically ill patients. Chest.

[2] Herget-Rosenthal S, Marggraf G, Hüsing J (2004). Early detection of acute renal failure by serum cystatin C. Kidney Int.

[3] Dinarello CA (2007). Interleukin-18 and the pathogenesis of inflammatory diseases. Semin Nephron.

[4] Award AS, El-sharif AA (2011). Curcumin immune-mediated and anti-apoptotic mechanisms protect against renal ischemia/reperfusion and distant organ induced injuries. Int Immunopharmacol.

[5] Luo Q, Zhou F, Dong H (2013). Implication of combined urinary biomarkers in early diagnosis of acute kidney injury following percutaneous coronary intervention. Clin Nephrol.

[6] Zheng J, Xiao Y, Yao Y (2013). Comparison of urinary biomarkers for early detection of acute kidney injury after cardiopulmonary bypass surgery in infants and young children. Pediatr Cardiol.

[7] Sirota JC, Walcher A, Faubel S (2013). Urine IL-18, NGAL, IL-8 and serum IL-8 are biomarkers of acute kidney injury following liver transplantation. BMC Nephrol.

[8] Stroup DF, Berlin JA, Morton SC (2000). Meta-analysis of observational studies in epidemiology: a proposal for reporting meta-analysis of observational studies in epidemiology (MOOSE) group. JAMA.

[9] Whiting PF, Rutjes AW, Westwood ME (2011). QUADAS-2: a revised tool for the quality assessment of diagnostic accuracy studies. Ann Intern Med.

[10] Rosman AS, Korsten MA (2007). Application of summary receiver operating characteristics (sROC) analysis to diagnostic clinical testing. Adv Med Sci.

[11] Li Y, Fu C, Zhou X (2012). Urine interleukin-18 and cystatin-C as biomarkers of acute kidney injury in critically ill neonates. Pediatr Nephrol.

[12] Chen TH, Chang CH, Lin CY (2012). Acute kidney injury biomarkers for patients in a coronary care unit: a prospective cohort study. PLoS ONE.

[13] Parikh CR, Devarajan P, Zappitelli M (2011). Postoperative biomarkers predict acute kidney injury and poor outcomes after pediatric cardiac surgery. J Am Soc Nephrol.

[14] Parikh CR, Coca SG, Thiessen-Philbrook H (2011). Postoperative biomarkers predict acute kidney injury and poor outcomes after adult cardiac surgery. J Am Soc Nephrol.

[15] Endre ZH, Pickering JW, Walker RJ (2011). Improved performance of urinary biomarkers of acute kidney injury in the critically ill by stratification for injury duration and baseline renal function. Kidney Int.

[16] Liangos O, Tighiouart H, Perianayagam MC (2009). Comparative analysis of urinary biomarkers for early detection of acute kidney injury following cardiopulmonary bypass. Biomarkers.

[17] Washburn KK, Zappitelli M, Arikan AA (2008). Urinary interleukin-18 is an acute kidney injury biomarker in critically ill children. Nephrol Dial Transplant.

[18] Parikh CR, Mishra J, Thiessen-Philbrook H (2006). Urinary IL-18 is an early predictive biomarker of acute kidney injury after cardiac surgery. Kidney Int.

[19] Parikh CR, Abraham E, Ancukiewicz M, Edelstein CL (2005). Urine IL-18 is an early diagnostic marker for acute kidney injury and predicts mortality in the intensive care unit. J Am Soc Nephrol.

[20] Lau J, Ioannidis JP, Schmid CH (1997). Quantitative synthesis in systematic reviews. Ann Intern Med.

[21] Zelenina M, Li Y, Glorieux I (2006). Urinary aquaporin-2 excretion during early human development. Pediatr Nephrol.

[22] Nash MA, Edelmann JRCM (1973). The developing kidney. Nephron.

[23] Dharnidharka VR, Kwon C, Stevens G (2002). Serum cystatin C is superior to serum creatinine as a marker of kidney function: a meta-analysis. Am J Kidney Dis.

[24] Parikh CR, Jani A, Melnikov VY (2004). Urinary interleukin-18 is a marker of human acute tubular necrosis. Am J Kidney Dis.

[25] Melnikov VY, Faubel S, Siegmund B (2002). Neutrophil-independent mechanisms of caspase-1- and IL-18-mediated ischemic acute tubular necrosis in mice. J Clin Investig.

[26] Tschoeke SK, Oberholzer A, Moldawer LL (2006). Interleukin-18: a novel prognostic cytokine in bacteria-induced sepsis. Crit Care Med.

